# Diabetes Management Using a Patient Navigator in a Native Hawaiian Population: Experiences and Perceptions of the Kilolani Project

**DOI:** 10.3390/ijerph22010060

**Published:** 2025-01-03

**Authors:** Nani L. Morgan, Nina A. Lopez, Amanda T. Campbell, Marguerite Cazin, Lauralee U. Ana, Jennifer F. Lai, May Vawer, James Yess

**Affiliations:** 1 The Queen’s Health System, Honolulu, HI 96813, USA; ninamurrow@gmail.com (N.A.L.); atcampbell1997@gmail.com (A.T.C.); lana@queens.org (L.U.A.); mvawer@queens.org (M.V.); jyess@hawaii.edu (J.Y.); 2 Department of Medicine, John A. Burns School of Medicine, University of Hawai’i at Manoa, Honolulu, HI 96822, USA; mcazin@hawaii.edu; 3 University of Hawai’i Cancer Center, Honolulu, HI 96813, USA; jlai@cc.hawaii.edu

**Keywords:** diabetes management, patient navigator, community health worker, Native Hawaiian, Indigenous people, Kilolani Project, health disparities, qualitative

## Abstract

Native Hawaiians (NHs) are a historically oppressed population disproportionately burdened by diabetes and related complications. The Kilolani Project, a patient navigator-centered, chronic disease management program, targets upstream drivers of health among vulnerable NH adult patients with diabetes within an urban academic safety-net clinic. To investigate the impact of the Kilolani Project, we performed a qualitative study to examine patient perspectives. Our goal is to ensure that their values, needs, and priorities drive future navigator-based strategies and healthcare delivery. Fifteen current Kilolani Project patients participated in one of three focus group sessions. Each session followed a semi-structured format with open-ended questions regarding their experiences with the Kilolani Project, patient navigators, and healthcare in general. Iterative transcript readings and coding revealed seven core themes: (1) Humanistic approach, (2) Trusting relationships, (3) Improved access, (4) Trauma-informed care, (5) Self-efficacy, (6) Resilience, and (7) Ethnic concordance. The Kilolani Project created a safe and culturally relevant experience for NH patients. They felt seen, heard, and valued, which resulted in an improved healthcare experience, engagement, and empowerment. Importantly, trust was the cornerstone to building a provider–patient relationship. Such culturally informed approaches are necessary to close the gap in health disparities faced by our NH communities.

## 1. Introduction

Type 2 diabetes disproportionately affects people of color and Indigenous populations across the United States (US), including those of Native Hawaiian and other Pacific Islander (NHPI) descent [1,2,3,4]. In the US, NHPIs are nearly twice as likely as the general population to receive a diabetes diagnosis [4,5]. Further, NHPIs experience more severe diabetes manifestations; they are at a higher risk for diabetes-related preventable hospitalizations, diabetes-related end-stage renal disease, and myocardial infarction [5,6]. Many of these disparities stem from the lasting impacts of colonization and well-recognized structural and social determinants of health, which have left a legacy of skepticism and deep-seated mistrust toward Western medicine [7,8].

Structural and social determinants of health are social, physical, and economic barriers that can impact an individual’s health-seeking behavior and well-being. These barriers include cultural and historical trauma, as well as perceived racism and discrimination resulting from the colonization of Hawai’i by foreign explorers who introduced infectious diseases that nearly decimated the entire, once-healthy Native Hawaiian population and forcefully seized ancestral land to exploit its natural resources for commerce [9]. These injustices undermined traditional subsistence practices due to forced relocation, and gave rise to the social and health disparities seen today. Compared to the general population, NHPIs experience higher poverty rates, lower educational attainment, and a higher prevalence of diabetes-related risk factors including poor sleep, poor nutrition, and food insecurity [5]. Moreover, studies report NHPIs to have low adherence to diabetes self-management, medication, and education participation [5].

Community health worker interventions have improved diabetes management in Indigenous and disadvantaged populations [10]. Community health workers, including patient navigators, are trusted, established members of a community. Oftentimes they are ethnically and/or contextually concordant with the people they serve and provide a culturally tailored coordination of social and health-related services that include culturally appropriate health education, informal counseling, coaching, social support, and advocacy [11,12].

Diabetes management programs led by patient navigators have succeeded in other minority and underserved US populations [13]. So have culturally appropriate, community-based diabetes education curricula in NHPI populations in Hawai’i [5,14,15]. However, there is limited evidence on using a patient navigator to manage diabetes at an individual level in Native Hawaiians [16]. And no qualitative studies have examined patient feedback regarding the use of a patient navigator.

The aim of this study was to examine the experiences and perceptions of Native Hawaiian patients with diabetes currently enrolled in the Kilolani Project, a patient navigator-centered diabetes management program in Honolulu, Hawai’i. Obtaining patient feedback is integral to ensuring that cultural and community derived-values and beliefs are incorporated into future program expansion and used to inform other patient navigator-driven intervention strategies.

## 2. Methods

### 2.1. Program Description

The Kilolani Project is a diabetes management program based out of an urban, academic, safety-net clinic in Honolulu, Hawai’i. The program aims to promote positive healthcare engagement and reduce barriers to care among vulnerable Native Hawaiian patients with diabetes. Every enrolled patient receives care at Queen Emma Clinics and consented to participate voluntarily without additional incentive. Comprising the Kilolani Project is a multi-disciplinary team consisting of a dedicated registered nurse, clinical pharmacist, and a Native Hawaiian patient navigator, who the program is centered around. Since its inception in 2021, the program has assisted 314 patients through various activities via phone or in person at the clinic, at home, or in the community. These activities include appointment reminders, follow-through on outstanding sub-specialty referrals, referral to social work and community resources, and health education and lifestyle management counseling. Importantly, unbound by specific agendas or time constraints, the patient navigator has strengthened *pilina* (connection or relationship) with patients by ‘talking story’, an important trust-building element in Hawaiian culture [16]. The registered nurse conducted the initial intake assessment to identify the patient’s clinical and psychosocial needs, while the clinical pharmacist (also a Certified Diabetes Care and Education Specialist) was responsible for medication reconciliation and adjustments. The patient navigator received approximately one month of training including the American Diabetes Association—Community Health Worker Diabetes Education Program as well as in-house cultural training with the institution’s Department of Native Hawaiian Health, which included sessions on trauma-informed care and historical contextualization. Additionally, the navigator shadowed social workers and patient navigators from three other programs within the institution targeting similar high-risk patient populations. The word “Kilolani” refers to a skilled navigator who uses traditional knowledge to wayfind across the open ocean using stars and other natural elements as guides. Similarly, the patient navigator helps patients wayfind through the complex healthcare system, using patient-centered values and needs as a compass.

### 2.2. Participants

Fifteen Native Hawaiian patients with diabetes enrolled in the Kilolani Project were recruited through convenience sampling to participate in one of three focus groups. Participants were identified and approached via a warm-hand-off during their clinic visits at Queen Emma Clinics in Honolulu, Hawai’i by their primary care provider, and through phone outreach. This study was approved by the University of Hawai’i Institutional Review Board. All participants provided written consent prior to participation.

### 2.3. Procedures

At the start of each session, the facilitator introduced herself and the study team. She then gave a brief overview of the study and explained the overall goal: to ‘talk-story’. The aim was to understand how Kilolani has helped them and how the study team can do better in the future. Reinforced was the fact that their voices and insight mattered, and that honesty was important and welcomed. Language was kept colloquial with local terminology included when appropriate. After the introductions, the patient navigator led an ice breaker, the purpose of which was to place the participants at ease with each other. Following this, housekeeping issues were addressed. Participants were assured there were no right or wrong answers; they should feel safe to speak openly in the focus group, while respecting each other’s views.

### 2.4. Measures

Three focus groups, with five participants each, were conducted in 2023 (one each in September, October, and November) at the Queen’s Medical Center in Honolulu, Hawai’i. Prior to each focus group, participants completed a brief demographic form that included multiple-choice questions regarding their experiences with Queen Emma Clinics and the Kilolani Project. Focus groups followed a semi-structured format guided by open-ended questions designed to elicit detailed experiences and perceptions regarding the Kilolani Project (Table 1). Focus groups were held on a weekday in a large conference room and lasted approximately 120 min without breaks. All sessions were facilitated by the Principal Investigator, a Native Hawaiian primary care physician (NLM). The Native Hawaiian patient navigator (LUA) and three study staff members were present to take notes and audio-record the interviews. Participants were offered transportation to and from the focus group if needed, lunch, and a $100 gift card for their time.

### 2.5. Analysis

Focus group discussions were transcribed verbatim immediately after each session using NVivo transcription software (Windows, Version 14). One team member then deidentified the transcript and fixed errors such as Hawaiian and Pidgin words, phrases, and names unrecognized by the transcription software.

Thematic analysis was used to review transcripts through a multi-step process involving reiterative readings and coding [17]. Three researchers (NLM, NAL, ATC) independently read and immersed themselves in the first focus group transcript, and then met to discuss and organize inductive codes into a codebook. A semantic approach of analyzing participants’ words at face value was employed. Following the initial codebook creation, four researchers (NLM, NAL, ATC, MC) reviewed and coded the second and third transcripts individually using Excel spreadsheets to organize participant quotes across themes. The researchers met on several occasions to compare findings. They discussed and resolved discrepancies based on group consensus and concluded their analyses when they agreed that theoretical saturation had been reached.

## 3. Results

### 3.1. Demographics

The 15 focus group participants ranged in age from 47 to 67 years (median 56.5) and consisted of seven females with four participants from rural areas. Nine participants were stratified as high-risk, five as medium, and one as low based on their assessed level of disease control and psychosocial barriers. As such, they received minimum monthly, trimonthly, and semiannual outreach, respectively, by the patient navigator or a Kilolani team member.

### 3.2. Summary of Themes

Seven key themes were identified (Table 2): (1) Humanistic Approach, (2) Trusting Relationships, (3) Improved Access, (4) Trauma-Informed Care, (5) Self-Efficacy, (6) Resilience, and (7) Ethnic Concordance.

#### 3.2.1. Theme 1: Humanistic Approach

- Aloha

Multiple participants acknowledged the importance of “aloha” as a way of life and an important factor in their broader healthcare and Kilolani experiences.

*“Especially for those of us who are born and raised here…that’s all we know is aloha.”*

Participants characterized “aloha” as *“a common courtesy for everything”* including basic respect and acknowledgement through verbal and non-verbal cues such as smiling and hugging.

*“Yeah, smiling helps you know what I mean? Laughter. All of that stuff helps everything. You know, it’s part of aloha.”*

*“We hug. No matter what the situation is, no matter what disease is out there, we don’t care. We just hug because that’s what gives life in each person is that hug.”*

- Compassionate Patient-Centered Care

Participants also described “aloha” as a deeper sense of being seen, heard, and valued by their providers, which contrasted with their prior healthcare experiences that lacked humanism, care, and compassion. In describing prior interactions with their providers, a participant shared, *“It’s so uh, robotic. You know, there’s no personal interaction, no one-on-one.”* Another participant similarly described experiences where *“[the doctors] just come in and think you’re an assignment on the computer.”* In elucidating these differences in approaches during clinic visits, this patient explained the following:

*“[The doctors] just come in and, you know, they’re smiling and they say hi. They’re not just there to do their job. You know, they know the person and they welcome the person, you know. And then they ask, how’s your day…you know, and then you respond to that, you know. But if you just come in and say oh, I’m Dr. So-and-so and…what’s wrong? You know, I was like, oh, what an aloha. Hello. You know, we’re in Hawaii. You know, everybody likes to be greeted instead of just going, oh, what’s wrong?”*

The same participant, a cancer survivor, later recalled how they once threatened to break the wrist of a healthcare worker who repeatedly jammed a nasogastric tube down their nose. *“…there’s tears coming down my face. I’m telling you that it hurts. It hurts. And [the healthcare worker] seem[ed] to not care, you know”.* Frustrated, they went on to describe,

*“Sometimes it’s just I feel like they think [of] their title because they have this white coat on, you know, and you’re supposed to do whatever they want to. You know, we’re human and we got feelings. We’re not just like some kind of boar you poke at.”*

When asked about their experiences with the Kilolani Project, participants reported feeling privileged to be part of a “special group” where they felt cared for and valued. *“[The Kilolani team] wanted to find out about me,”* one participant said. *“That’s what their goal was, not solving it yet, just they wanted to find out about me. What am I worried about?”*

#### 3.2.2. Theme 2: Trusting Relationships

Participants emphasized the importance of building *pilina* (connection or relationship) with their healthcare team. This was particularly important because, as one participant put it, “*Hawaiians is all about connection*.” Another participant went on to explain,

*“Because…for example, during our days, she from the mountain, he from the ocean. How we connect is by trading, exchanging. … So like patients to doctors or nurses. We share what we, you know, what we go through, and stuff like that, and in exchange you guys give us advice or, you know, whatever. So that’s the most important. Having a connection. Without the connection, no can.”*

Building *pilina* was the foundation from which trust was obtained, a topic emphasized throughout the sessions.

*“Because the trust. The trust. The trust is not there at first.”*

That said, the current system faces many challenges with regards to sustaining continuity of care, especially within a teaching health center where residents only serve on the primary care team for three years before graduating. Patients expressed frustration with having to start over with their primary care team members; they described repeatedly having to answer the same questions and often having decisions made for them without a caring relationship in place.

*“I understand [the Queen Emma Clinic] is a learning place…You got to start someplace…But [the residents] go straight to the beginning. I been here three years ago and you asking me a two-year-old question.”*

*“Here we go again. Same question. Over and over.”*

*“And [the residents] say, ‘Oh, we’re taking this medicine off and we’re doing this.’ And I’m like, ‘Wait a minute, go away.’…”*

*“I didn’t like to keep repeating myself over and over with different residents, the same thing over and over. And I got tired of that. …They said that they going increase my medicine if I say something, you know what I mean? And I just met them and that’s the part that I didn’t like about them, that they would say, ‘Oh, I think we have to do this. We have to do that.’ And I looking at them, I like to be polite, yeah? And I just say, ‘Um, I no think so.’”*

In addition to sharing frustrations about poor continuity among their providers, the frequent turnover of clinic support staff was also a source of frustration for some.

*“I’m so used to when you come in, everybody goes hi. And then [one time] I come in and I’m about to say hi and then they going, ‘who are you?’ The reception is all different. There’s not the same people you see all the time. And then I’m like, ‘who are you?’ and they start asking me all these questions. I go, ‘no you didn’t answer my question. Who are you?’”*

Despite these challenges in building *pilina* with their healthcare team, participants valued having a familiar and dependable member to call on. In speaking about the patient navigator, a participant shared, *“… it’s nice to know that you have someone you can call, and you can lean on her.”* Participants described a strong bond with the patient navigator to the extent that she was even viewed by some as *‘ohana* (family).

*“And, you know, she’s like more of a sister than a worker because you can call her any time and say hey…I got this problem, you know, like it’s not only [that], we’re not talking about things that happen here. We’re talking like the whole thing about my life, you know? I say, can you help me with other stuffs? And she’s right there.”*

For some, *pilina* did not happen right away. One participant remembered their initial reservation about the navigator’s persistence after repeated questioning of her purpose, what she was doing, and why she was there.

*“[The navigator] keep telling me, ‘oh I’m here for you’ and stuff like that. And I’m thinking, wow, I don’t need this. …But then I always ask her, ‘Why are you here?’ And she goes, ‘I’m here for you.’ One day I ask her, ‘why are you here?’ She goes, ‘I find you interesting. And it was because she kept being there. And then it was like, I gotta let this girl in. And then when I get problems, I just call her. Every time.”*

Another recalled how their upbringing taught them not to trust and to be defensive right away. If someone offered to help, something was wrong, or they wanted something from you. After finding trust in the navigator, they noted, *“to find people that really understanding [sic] where you’re coming from, you know, [that’s] what you need.”*

#### 3.2.3. Theme 3: Improved Access

- Availability

Participants appreciated the navigator’s availability; they could call anytime and avoid past delays in reaching the clinic.

Frustration was expressed regarding contacting staff and scheduling appointments. One participant explained,

*Well first you gotta go to that operator who keep telling you what’s going on. Then you get to the reception. They gonna take you around the block [murmurs of agreement]. But the reception, they’re gonna have no idea what you’re talking about. Even if they just called you! You know, you missed that call so you call back, ‘oh you guys just called?’[they reply] ‘oh I don’t think so.’ I’m going ‘you guys just called!’”*

However, within the Kilolani Project, patients were appreciative of being able to call the patient navigator directly for any needs. *“I don’t even call the clinic anymore, I just call [the navigator].”* Because the navigator knew the patients, their circumstances, and barriers to care, she was able to accommodate their scheduling needs. One participant traveling from the westernmost side of the island (often a two-hour commute) said,

*“It’s like this hour battle there with Queen Emma Clinic…they’re scheduling us like 2:30–3:00 appointments and we come from Waianae. Now we don’t travel, you know, especially back home in traffic. And that’s what our thing was. So, you know, I say, if you schedule us then, we’re not coming. So, you know, I let [the navigator] know if I’m gonna come, gotta be [there] earlier. So that’s what she helped us with.”*

- Health-Related Social Needs

Also appreciated was the navigator’s ‘open’ approach, her willingness to help with non-clinical issues such as completing paperwork, transportation, financial issues, and housing among others. *“You can just go in and talk story and be, hey, you know, anything you can talk about and they’ll help you. If they don’t know, they will find out for you,”* one participant said, *“it just feels that you can come there any time and you can, they’re dependable.”*

Another shared gratitude for help in obtaining food stamps and social security disability:

*“They sent somebody over from Kilolani and basically he sat down and he just talked with me, things that I’m needing, things I’m worried about. So I didn’t even know I qualified for food stamps because I quit my last job. And then I did that before. But when I called them up, they said, no, you got to wait six months since you quit. But [the Kilolani staff member] was like, No, no, no. So he did it on the computer with me on the phone for a while in the emergency room. Got the SSDI on there also. I mean, every little bit helps.”*

Another patient recalled an instance when a Kilolani staff member helped them when their transportation failed to show and they had no phone. *“The first thing is I told myself, I’m going to go Kilolani and talk to the Hawaiians. Cause they understand.”* After explaining their situation, the staff member sat them down and then went inside to follow up. *“[The Kilolani staff member] came back out and she told me, ‘Your car is, uh, going to be 15 min late, but it’s coming’…. So, you know, I’m very grateful that I can narrow it down to one person or somebody in particular that’s working to help me in this situation.”*

After being admitted to the hospital for acute cardiac issues after waiting in the emergency department for 16–18 h, another participant recalled being concerned about their moped being towed. They called the patient navigator, who immediately allayed their worries by helping to move their moped to a secure place for the night.

- Persistence

Participants valued the navigator’s routine outreach, multiple calls to remind them of incomplete labs and appointments, and general persistence. One participant reported, *“They hound you like [laughter] if you miss your appointment. They going make sure, you make another one….You cannot run away.”* Another shared their appreciation by saying,

*“You know, and um, tell me what I need or try keep me updated and you know, with what I gotta do and stuff in the clinic. Yeah, so uh without [the navigator], you know, letting me or reminding me because of all these situations like I have, um, I think I’d be lost, you know? But with her, she always kinda keep me in line.”*

#### 3.2.4. Theme 4: Trauma-Informed Care

Multiple participants spoke about the importance of mental health in the face of prior trauma and associated stigma. One participant who had struggled off and on with homelessness shared,

*“You never know what happened to people, why they on the street or, you know, what kind of bad things happened or what they saw in front of them or what they was given, you know? So mental health is a big issue.”*

- Histories of Trauma

A participant from Papakolea (an urban Native Hawaiian homestead) spoke of a time when the neighborhood boys would roam past, drinking and smoking weed. *“Actually they called me a tita (tough woman) because I used to beat up the boys. I had no choice…”* They knew what the boys could do to them. “*So we decided, the women in Papakolea decided you know what, we all get together. These guys gonna start something. Let’s beat the shit out of them. So that way they’ll leave us alone…. So we had no choice. We have to defend ourselves*.”

The same participant opened up about prior physical abuse by family members, *“I’ve been shot. I’ve been stabbed. I’ve been thrown out of a window by my own family. That’s the thing you know. We all grew up differently. And we all went battle things as we grew….We don’t go do bad things. We just hurt because what our family do to us.”*

A few participants spoke about cultural trauma; one participant did not learn about their family’s history of being traditional Hawaiian healers for the *ali’i* (Hawaiian royalty) until after their cousin died of stomach cancer in a hospital. They recalled,

*“I wasn’t taught that when I was raised. I was raised Portuguese, my grandma. The Hawaiian side was diminished from us. Was actually like being black and white in my days. My mom was banished, like, you know what I mean? Had to wait in the room for when the family, come over, go eat…. It goes deep, man.”*

Another participant, who grew up exclusively speaking *‘ōlelo Hawai’i* (Hawaiian language), recounts the loss of their native tongue.

*“Eventually we didn’t speak Hawaiian for a long time. And so going to church, what helped us is that my grandfather was just because he knew that us grandchildren was learning English in education-wide….I did grown up with Hawaiian, but it’s been so much years that I forgot.”*

- Mental Health Stigma

Despite these ubiquitous trauma histories, few sought help due to the stigma surrounding mental health. Such stigma, instilled in early childhood, caused participants to suppress their emotions well into adulthood.

*“Mental health was a weakness. Cannot be weak, right? Gotta be strong. Cannot tell your shit to nobody. When you finally tell somebody. Most of them don’t listen anyway, you know. So why are you going to tell them anything?”*

*“What crying going do? Crying only going get you more lickings. ‘I give you something to cry about’, pow!”*

*“We were taught…what happens in the house, stays in the house…nobody’s problem but yours. But when it builds in your head so long, you just want to explode and you don’t know who to turn to because you were taught not to trust.”*

Another participant shared frustrations about the stigma associated with mental health diagnoses that prevented them from wanting to seek care.

*“It shouldn’t be a label. That’s why people are reluctant to come to doctors because they get labeled…I was labeled as manic depressive [at] 14 years old. You know what I mean, not knowing what is that and then the guy’s like the guy diagnosed me right there in two minutes. Like brother, how you did that… I’ve been through rough childhood… I’m fine now.”*

In reflecting on their prior trauma and the stigma they faced, many participants had come to appreciate the centrality of mental health in their overall sense of well-being. As one participant said, *“If you not mentally right to take care of yourself, you not, okay?”* The participant continued, *“I don’t care how physical shape you are, if your mental health not working. You are not. You are just not going [to] do anything happily.”*

- Kilolani Support

Fortunately, the *pilina* participants had developed with the navigator allowed them to talk more openly about their trauma and daily stress in a way they had never done before.

One patient was struggling to process previously suppressed childhood trauma, feeling as though they had been *“[thrown] out the plane without one parachute with no tools to deal with it”.* They recalled the navigator throwing them *“a lifeline.”* They explained, *“I called [the navigator] up again….this was all within five days, I was calling her every day. And every time I talked to her, I got better and better….that was the lifeline.* They went on to say, *“…if [it] wasn’t for [the navigator] I would be still hanging by a thread. That thread would have broken by now….I never knew mental health was an issue. And it is. You get all the shit kicked up. Your past comes back. Things that you buried down inside come up. I thank you [addressing the navigator] for that many times. Made me laugh when I wanted to cry.”*

Kilolani served as a social support system that allowed many to cope with stress. Raised illiterate, one participant disclosed not attending school, being pulled out in eighth grade by their mother due to trauma endured at home. They said, *“…and, you know, [the navigator] knew about that. So every time when I just need to talk, you know, she’s there.”*

Another shared stressors related to living with their brother, who struggled with alcohol use disorder.

*“…when it’s really stressing me out…[the navigator] helps me, she’s just there, listen then, you know….[You] go from there” They went on to say, “You gotta have somebody you can talk to. Not really yell and swear. But just open.” They continued, “God, I can call her like 5:30 in the morning, she stay working already… I ready [to] call 988 almost, you know. […] Yeah. The suicide hotline. …I’m stressed that much and I was like, I’m [going to] call [the navigator]. And she answers….[she’s] very compassionate. Very understanding. Listens.”*

#### 3.2.5. Theme 5: Self-Efficacy

- Personal Responsibility

Multiple participants felt a strong sense of personal responsibility for their health.

*“You got to do your part so you can get better, and all that stuff.”*

*“If somebody telling me something wrong with me, it’s up to me to fix that.”*

*“The medication is medication, but dedication is more better. Because you got to be dedicated for your health, you know… health is wealth.”*

*“And all you want to know is why. You know…this is your body. You gotta walk around with it you know. So, you want to know what’s [sic] the hell going on in here? How can I fix it? How can I fix it?”*

- Empowerment through Education

Participants expressed interest in learning about diabetes and how to better care for themselves. One said,

*“I remember [the] doctors say, ‘Oh, you got this and that, take that. Pau (finished).’ And you’re like, What? …there’s so much education, the doctors are even explaining more…. it makes me want to know more because, wow, I didn’t know that my body did this and this and that. You know, and now it’s like, okay…this is what I can do…. So… I want more.”*

Another participant expressed a new sense of empowerment in learning how to advocate for themselves by asking questions of their providers,

*“…when you growing up, you listen to your parents and stuff. Whatever the doctors say, that’s what it is….when we were younger, we didn’t know we could ask them questions. Like they say ‘you have any questions,’ but, like, what kind of questions?… learning more give[s] each individual more power to their self….if they ask questions, then the doctor can help them, then they feel better about themself. Because you in control.”*

One participant expressed feeling devastated, thinking their new diagnosis of diabetes was a life sentence. *“I was diagnosed with diabetes when I was about 40 something. And I thought, when I first found out that I was diagnosed with that, I thought my life was kind of, like, ending. Didn’t know anything about diabetes…I was just thinking, ‘Oh my God, I wonder if I going live long.’”* However, through positive healthcare engagement and education, they were able to improve their confidence. *“But when I started coming, you know [to Queen Emma Clinic], when I heard about classes they had here, uh I came. And I came to every one that they had to kind of get me to understand my diabetes, and all of that. And like, now today, I feel really good about it.”*

The Kilolani Project helped patients better understand their condition, and motivated them to improve their health behaviors. One participant shared,

*“I learned a lot. A lot about health and nutrition in the last year or so. And I’ve been talking to [the navigator] a lot about it. She said like, ‘Wow, you so awake now,’ you know what I mean?”*

Another experienced a renewed sense of optimism in working with the Kilolani team:

*“And then when I see the numbers. Like every month, when the thing is declining, I’m like, wow. I thought I would never, ever get it down.”*

In advising about future directions for the Kilolani Project, multiple participants wanted to expand opportunities for continued education and support: *“We’re telling you that. We’re telling you need…support groups so people can understand what’s going on…we’re going try and find out how to help ourselves”*

Another participant stated, *“…getting up there in age, all these health problems, you know, well, not health problems, but…you know, body going through so much that I…I want to understand, get more information. Find out more to help me actually navigate it…Yeah.”*

For some, the desire for education extended beyond themselves. They wanted it for their families and community. *“You know, you never stop learning. You know, you can learn more and not only just learn for yourself, but you can share it with your family and you can share it with, you know, your friends and everybody that you can come in contact with.”*

#### 3.2.6. Theme 6: Resilience

Several participants expressed a strong sense of resilience, which they credited to their cultural traditions and spirituality. One participant proudly asserted, *“That’s what locals [are]. Hawaiians. Brothers, sisters. We survivors, man. No matter how you look at it. If you need to survive, look at Hawaiians…You know how fish?… Have you ever been in a mountain and gutted a pig?”*

Participants spoke of traditional knowledge or life skills that had been passed down from their ancestors as sources of strength and pride, yet not always recognized by traditional Western perspective.

*“So my mother really never went to school, but she learned from her kupunas (elders), and what she learned, I have learned. And so I never went to school for playing, how to play ukulele, keyboard, or sewing. And yet I can do that today.”*

Many acknowledged their connection to the *‘āina* (land) as a source of vitality through practices such as fishing, diving, hunting, and farming, among others.

*“I surf every day. Every morning, early in the morning, like five o’clock … But my favorite part of that is watching the sun come up over Diamond Head. Every day. Even just thinking about that, it’s just like the possibilities of a new day.”*

*“I’m doing lauhala work [weaving art form using leaves from the Hala or Pandanus tree]. I do lauhala visors. Like this. Just to bring our ancestors back to say, you know what ancestors? I’m doing what you guys asked me to bring you guys back.”*

A few participants shared how they called on God for strength. One reported, *“I mean, I can have all the problems, all the stress, everything. But when you actually sit down and talk to God. It takes everything off of you and it works for me.”*

Shaped by ancestral wisdom and having the capacity to pass it forward was also important to some. *“That we learned from our grandparents and what we learn, we teach to the next generation. What they learn, they teach to the next generation.”*

#### 3.2.7. Theme 7: Ethnic Concordance

Participants were mixed on ethnic concordance. Some felt that having a navigator with Native Hawaiian blood or ancestry was not necessary, but felt it was helpful to have someone “local” (born and raised in Hawai’i) with cultural familiarity.

One participant shared the importance of shared communication style.

*“You got to speak to them in our language…You just got to learn Pidgin…The locals, I mean lot of the locals here, they’re willing to open up, they’re willing to learn new things, but come down to where our level is…got to be at our levels brah.”*

Another shared that having someone local was important for trust, stating,

*“You cannot trust somebody else. You know, we have a local person. They know what we been through.”*

Reflecting on a prior experience with a foreign physician, one participant noted,

*“He was alright, but it’s hard for somebody to come from another country to learn how we as people here local, you know, adapt to things, you know.”*

To other participants, having a similar ethnicity mattered less than other qualities, like a caring personality and attitude. As one participant stated,

*“It could be anybody… not because of what their race is… it’s how…they project themselves.”*

Another shared,

*“Whatever they have in their hearts.”*

One participant recalled a time when their husband had surgery,

*“The guy who did the surgery had talked to me. Said, ‘don’t worry.’ And he was really nice, really polite. And he was a haole (Caucasian). And I looked at him. I said …where did you get this kindness, love, respectful [sic]. ‘Oh,’ he goes, ‘oh, my grandmother.’ That’s how it’s supposed to be. I don’t care what nationality you are…. I gave him a hug…He goes, ‘why?’ I go, ‘because you taking care of my husband. And you’ve got good heart.’ That’s what I saw.”*

## 4. Discussion

Native Hawaiians have unique health needs. Due to past injustices, they require culturally responsive approaches that respect their Indigenous perspectives, which are often overlooked in Western medicine. Patient navigators can help Native Hawaiians as well as other Indigenous patients maneuver through the healthcare system, bridging cultural gaps to overcome barriers in accessing resources while attending to emotional needs. In doing so, they foster trust and empowerment in those they serve.

Our patient navigator-led Kilolani Project provided a safe and meaningful experience for Native Hawaiian patients living with diabetes. Patients valued the navigator for treating them with *aloha* (reciprocal love), which they described as feeling respected, cared for, and valued. This sense of *aloha* strengthened *pilina* (connection or relationship) to the extent that they came to see the navigator as ‘*ohana* (family), someone they could rely on anytime, without judgment, to provide an empathetic and sympathetic ear for clinical as well as non-clinical issues. Though patients did not feel ethnic concordance was necessary, they found it helpful to have a “local” navigator with cultural familiarity along with someone who they had developed a trustworthy relationship with. Patients spoke more openly about their prior trauma, perceived stigma, and day-to-day stresses in ways they had never done before. And through these dialogues came resilience. Despite their troubles, patients found strength in religion and traditional beliefs, and they credited the navigator for helping them reclaim their health.

The findings from this project fit well within existing, culturally grounded health frameworks that view Native and Indigenous peoples from a holistic perspective, stressing harmony in physical, mental, and spiritual health. Grounded in the Native Hawaiian worldview, Kūkulu Kumuhana [18], for example, emphasizes well-being as an interconnected and collective responsibility that spans relationships, community, and *‘āina* (land). This framework diverges from Western health paradigms that often highlight individual success metrics and instead captures the broader, more relational aspects of well-being through six overlapping dimensions rooted in traditional values and *ʻōlelo Hawai’i* (Hawaiian language): (1) *Ea* (self-determination), (2) *ʻĀina momona* (kinship with and respect for nature), (3) *Pilina* (connection or relationship), (4) *Waiwai* (collective wealth and abundance), (5) *ʻŌiwi* (cultural identity), and (6) *Ke Akua Mana* (faith and spiritual strength). The Pilinahā framework [19], actualized through *moʻolelo* (storytelling) with community members of a similarly vulnerable population, highlights—as denoted by its name (*pili* meaning connection, ‘*ehā*, four, and *hā*, life)—the four vital connections for life. These include connections to (1) place (kinship with and access to the *ʻāina*), (2) community (reciprocal loving relationships), (3) past and future (possessing *kuleana*, responsibility and accountability for what we have control over), and (4) your better self (knowing oneself, being comfortable with who you are).

In line with “*pilina*” and “*connection to community*” highlighted in the frameworks above, the single most important component to ensure our patients felt cared for was developing a trusting relationship with the navigator. Only after trust was established could the navigator address the patients’ concerns. This is consistent with the experiences of rapport with other Indigenous populations [20]. The navigator formed this trust through her caring and compassionate approach; she listened deeply and regularly, and developed plans based on the patients’ needs and life stories, making them feel heard and understood. Likewise, “*ea*” and “*connection to your better self*” was mirrored by the patients’ self-efficacy; they expressed commitment to take charge of their health and to empower themselves through education. For some, this pledge went beyond themselves. They wanted their family and community to experience the wealth of knowledge bestowed on them by the navigator, reaffirming “*waiwai*”(finding value in intangible assets such as collective health). Working with the navigator instilled in patients the belief that health is an ongoing journey, not a fixed destination; their challenges do not determine their outcomes. As such, they opened up about past hardships, which allowed them to cope with current stresses and guide their next actions. This practice validated the “*past-future*” component of well-being. Patients valued the navigator’s role throughout this process, seeing her as a source of emotional support in the dual capacity of an adviser and confidant. Due to this *pilina* with the navigator, patients remained optimistic despite their circumstances. They found strength through cultural practices, ancestral wisdom, and God (*ka akua mana*), which helped them maintain their identity as Native Hawaiians (*‘ōiwi*).

### 4.1. Future Directions

The focus on efficiency, productivity, impersonal centralized services, and standardization in Western medicine continues to fuel Native and Indigenous peoples’ perceptions that healthcare lacks compassion, empathy, and availability. They are not wrong. Ironically, system-wide “patient-centered” initiatives often protocolize impersonal interactions and potential “touch-points”. Impersonal, nationalized “quality” goals and metrics regularly replace a more nuanced culturally informed means of ascertaining health status and trajectory. Findings from this study and others [21] highlight the desire for patient–provider connection and rapport. These attributes, however, run counter to reality. Patients often suffer from decreased meaningful clinical interactions due to increased productivity demands. Doctors have less and less time to make a qualitative impact, and the current system’s rigidity only adds to the many barriers already faced by patients.

To reduce health disparities in diabetes and other chronic diseases among Native Hawaiians and other Indigenous populations, healthcare providers must listen to their patients’ invaluable experiences and perspectives. Kūkulu Kumuhana and Pilinahā provide an essential framework for conceptualizing Native and Indigenous well-being and serve as a guiding philosophy for implementing effective, culturally aligned interventions such as the Kilolani Project to foster trust, resilience, and empowerment in Native Hawaiian patients. In this regard, patient navigators are the key. They mitigate institutional barriers to ensure equitable access and, for these reasons, should be integrated as part of the healthcare team, not just an adjunct.

Very recently, the Centers for Medicare & Medicaid Services (CMS) announced policy changes to include reimbursement for patient navigation services [22] that, for many years, relied on time-limited grants or institutional funds without third-party contributions. The new policy also expands coverage for hemoglobin A1c testing for pre-diabetes and diabetes screening, and diabetes management. We fully support and applaud CMS’s policy changes, which recognize the value of patient navigation as a mechanism for advancing health equality for Native and Indigenous communities. Future studies should assess the potential hospital cost savings and quality-of-life changes in patients and providers resulting from CMS’s policy updates.

### 4.2. Strengths

Focus groups were chosen for this study due to their effectiveness in capturing a wide range of perspectives and fostering rich discussions. For Indigenous populations, especially Native Hawaiians, *moʻolelo* (storytelling) is a vital form of data collection that honors cultural rituals by conveying individual experiences while embodying collective memory and traditional values, making it an invaluable method for understanding nuanced perspectives. Using *moʻolelo* ensured that collected data were culturally relevant and reflected the patients’ lived experiences, in addition to upholding the principles of respect, equity, and sovereignty key in research involving Native Hawaiians.

### 4.3. Limitations

There are a few limitations to the study. First, although common practice, participants were recruited for focus groups through convenience sampling. This may have resulted in the biased selection of participants with higher levels of engagement with the Kilolani Project and even the healthcare system as a whole. By offering transportation to and from focus groups, we were able to at least diversify our sample to include individuals with disabilities, transportation challenges, and those coming from more rural areas of the island. Second, having the patient navigator present throughout the focus group sessions may have biased participant responses in favor of the Kilolani Project. Nonetheless, we felt the navigator’s presence was essential in promoting more open conversation among participants who may have otherwise not been as forthcoming with staff they did not know. Third, although our modest sample size of 15 participants was adequate to achieve theoretical saturation, it might not accurately represent the opinions and experiences of every patient included in the Kilolani Project. Last, we acknowledge that everyone has their own implicit biases that may influence how data are interpreted and presented. To help address this issue, we intentionally included a team with diverse backgrounds and experience. Each team member coded the transcripts independently and the team met frequently throughout to discuss and resolve differences.

## 5. Conclusions

Our Native Hawaiian patients with diabetes benefited from patient navigation. They described feeling seen, heard, and valued, and appreciated the navigator’s willingness to help with non-clinical issues. Several traits were deemed important for healthcare providers. These included the following: empathy, compassion, respectfulness, dependability, and, most important, trustworthiness. Patients did not feel ethnic concordance was necessary, but found it helpful to have someone “local” with cultural knowledge. The main suggestion was to provide more education and support groups while considering culturally relevant ways to strengthen self-efficacy and resilience. This study’s results can be used to inform other patient navigator-led interventions and improve healthcare delivery with Native and Indigenous populations.

## Figures and Tables

**Table 1:** Focus group questions.

1	Describe your experiences with the Kilolani Project. What do you like about the program? What could the program do better?
2	In what ways does having the Kilolani community navigator make your healthcare experience different from previous experiences?
3	Describe a positive experience with the Kilolani community navigator. What made it positive?
4	Is it important to you that the Kilolani community navigator is Native Hawaiian? What qualities are important to you?
5	What are your top health priorities for yourself, your ‘*ohana* (family), or your community that you would want the Kilolani Project to address?

**Table 2:** Summary of themes with representative quotes.

Theme	Sub-Theme	Quotes
Humanistic Approach	Aloha	*“Yeah, smiling helps you know what I mean? Laughter. All that stuff helps. You know, it’s part of aloha.”*
	Compassionate Patient-Centered Care	*“That’s what their goal is, not solving it yet, just they wanted to find out about me. What am I worried about?”*
Trusting Relationships		*“…she’s more like a sister than a worker…”*
Improved Access	Availability	*“I don’t even call the clinic anymore, I just call [the navigator].”*
	Health-Related Social Needs	*“Anything you can talk about and they’ll help you. If they don’t know, they will find out for you…it just feels that you can come there any time and you can, they’re dependable.”*
	Persistence	*“They hound you like [laughter] if you miss your appointment…”*
Trauma-Informed Care	Histories of Trauma	*“I’ve been shot. I’ve been stabbed. I’ve been thrown out of a window by my own family. That’s the thing you know. We all grew up differently…”*
	Mental Health Stigma	*“We were taught…what happens in the house, stays in the house…nobody’s problem but yours. But when it builds in your head so long, you just want to explode and you don’t know who to turn to because you were taught not to trust.”*
	Kilolani Support	*“…and, you know, [the navigator] knew about that. So every time when I just need to talk, she’s there.”*
Self-Efficacy	Personal Responsibility	*“You got to do your part so you can get better, and all that stuff.”*
	Empowerment through Education	*“…getting up there in age, all these health problems, you know, well, not health problems, but…you know, body going through so much that I…I want to understand, get more information. Find out more to help me actually navigate it…Yeah.”*
Resilience		*“That’s what locals [are]. Hawaiians. Brothers, sisters. We survivors, man. No matter how you look at it. If you need to survive, look at Hawaiians…*
Ethnic Concordance		*“You cannot trust somebody else. You know, we have a local person. They know what we been through.”*

## Data Availability

Data are available from the corresponding author upon reasonable request.
